# Engineering a Virus‐Derived X Family DNA Polymerase FvPolX for *de novo* DNA Synthesis

**DOI:** 10.1002/advs.75017

**Published:** 2026-03-31

**Authors:** Chengjie Zhang, Lei Du, Shengying Li

**Affiliations:** ^1^ State Key Laboratory of Microbial Technology Shandong University Qingdao Shandong China; ^2^ Laboratory for Marine Biology and Biotechnology Qingdao Marine Science and Technology Center Qingdao Shandong China

**Keywords:** 3'‐ONH_2_‐dNTPs, *de novo* DNA synthesis, enzyme engineering, X family DNA polymerases

## Abstract

Enzymatic *de novo* DNA synthesis has gained increasing attention over the past decades, having emerged as a hotspot in synthetic biology. However, current research remains largely focused on animal‐derived X‐family DNA polymerases (PolXs), such as the extensively studied terminal deoxynucleotidyl transferases (TdTs) from vertebrates and the recently identified RvPolX from the invertebrate *Ramazzottius varieornatus*. In contrast, microbial PolXs have largely been overlooked, despite their potential in catalytic diversity and industrial applicability. Here, we report the discovery and rational engineering of FvPolX, a PolX enzyme derived from *Faustovirus*. Through protein engineering, we developed the FvPolX^R184L/T186G/N267S^ variant, which demonstrates a dramatic enhancement in catalytic activity toward both canonical dNTPs and noncanonical 3′‐ONH_2_‐dNTPs—transforming the enzyme from nearly inactive to highly efficient. Its template‐independent DNA synthesis efficiency surpassed that of wild‐type TdTs and is comparable to that of engineered TdT variants for adding 3′‐ONH_2_‐dNTPs. Additionally, we constructed a truncated version of FvPolX (s149FvPolX^R184L/N267S^) containing only the palm and thumb subdomains. This minimal double mutant retained high catalytic activity in template‐independent DNA synthesis and efficiently incorporated both canonical dNTPs and 3′‐ONH_2_‐dNTPs. Together, these findings expand the enzymatic toolbox for *de novo* DNA synthesis by diversifying polymerase sources and exploring structural minimization.

## Graphical abstract

In this study, a *Faustovirus*‐derived polymerase (FvPolX) is engineered based on structural and sequence analysis of PolX enzymes. The optimal mutants demonstrate substantially enhanced catalytic activity toward dNTPs and 3′‐ONH_2_‐dNTPs compared to the wild‐type enzyme. This work expands the enzymatic toolbox for *de novo* DNA synthesis by diversifying polymerase sources and exploring structural minimization.

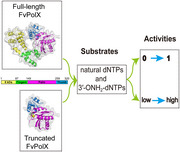

## 1 Introduction

Enzymatic DNA synthesis has garnered significant attention and achieved remarkable breakthroughs in the past decades [1, 2, 3, 4, 5, 6, 7, 8, 9, 10, 11]. This important enabling technology holds tremendous potential for a broad range of applications across diverse fields, including gene/genome synthesis [12, 13, 14, 15], DNA‐based data storage [16, 17, 18, 19, 20, 21], DNA origami [22, 23, 24, 25], medical diagnostics [26, 27, 28, 29, 30, 31, 32], and environmental monitoring [33, 34, 35, 36]. To date, the research on enzymatic DNA synthesis has been focused on terminal deoxynucleotidyl transferases (TdTs), the primary enzyme used for template‐independent nucleotide polymerization. Recent studies on TdTs mainly include enhancement of catalytic activities toward natural and unnatural substrates [6, 10, 37, 38, 39, 40, 41, 42], improvement of thermal stability [9, 43], and optimization of protein expression levels [44].

TdT belongs to the X‐family DNA polymerases (PolXs). However, enzymatic DNA synthesis involving other PolX family members remains largely unexplored. PolXs are widely distributed across the biosphere, including eukaryotic, bacterial, archaeal, and viral organisms [45, 46, 47, 48, 49]. These enzymes mainly participate in DNA‐repairing processes through both template‐dependent and template‐independent mechanisms. Mammalian PolXs—including TdT, Pol *λ*, Pol *µ*, and Pol *β*—play central roles in base excision repair (BER) [50, 51, 52], non‐homologous end joining (NHEJ) [53, 54, 55, 56], V(D)J recombination [55, 57], and double‐strand break repair [58, 59]. Recently, a PolX enzyme from the extremotolerant invertebrate *Ramazzottius varieornatus* (RvPolX) was biochemically characterized by Law et al. [8]. Through saturation mutagenesis of 12 residues within its dNTP‐binding pocket, followed by high‐throughput screening, a double‐mutant (G513A/R522I) was constructed with significantly enhanced activity toward all four natural dNTPs—most notably, showing a ∼35‐fold improvement in dATP incorporation over the wild‐type enzyme [8].

In contrast, bacterial and archaeal PolX enzymes remain underexplored, despite their great diversity. Bacterial PolXs include canonical (e.g., *Thermus thermophilus*, *Bacillus subtilis*) and noncanonical variants (e.g., *Deinococcus* spp). These enzymes are often involved in the NHEJ pathway and may function in the double‐strand break repairing process or BER [60, 61, 62, 63]. Noncanonical bacterial PolXs usually lack DNA polymerase activity due to divergence in the catalytic triad (typically composed of three conserved acidic residues) [63]. Nevertheless, they display strong 3'→5' exonuclease activity (probably also with proofreading function) via their highly conserved C‐terminal exonuclease‐polymerase and histidinol phosphatase (PHP) domains, which is unique to prokaryotic PolXs [64, 65, 66, 67, 68]. PolX enzymes of archaeal origin have yet to be comprehensively characterized.

Among viral PolXs, the African swine fever virus PolX (AsfvPolX) represents the most studied one to date [69, 70, 71, 72, 73]. With a minimal molecular mass of approximately 20 kDa, it is the smallest known DNA polymerase, which has no proofreading 3'→5' exonuclease activity [74, 75, 76]. Unlike intact PolX core domains (PolXc), AsfvPolX lacks both the 8 kDa and fingers subdomains, retaining only the palm and thumb subdomains responsible for catalysis and substrate binding. The 8 kDa and thumb subdomains typically interact with DNA substrate via helix‐hairpin‐helix (HhH) motifs (GhG/Axxxx) [49, 64, 77]. And the 8 kDa subdomain contains the residues responsible for the 5'‐deoxyribose phosphate (dRP)‐lyase activity potentially important for their functions in BER [78]. The amino acids necessary for dRP lyase activity are not conserved in eukaryotic PolXs, and Pol *μ* and TdT possess an inactive 8 kDa domain. Two conserved dNTP‐binding motifs are located within the palm and thumb subdomains (residues ^179^GSFRR^183^ and ^271^YFTGSDIFN^279^ in human Pol *β*), mediating interactions with dNTPs via hydrogen bonds and van der Waals forces [63]. AsfvPolX functions in coordination with other viral DNA repair enzymes, including apurinic/apyrimidinic (AP) endonuclease and DNA ligase (AsfvDNAL) [79].

Expanding the phylogenetic diversity of polymerase sources is a promising strategy for biocatalyst engineering. While significant efforts have been devoted to TdT optimization for practical gene synthesis, the *de novo* DNA synthesis potential of non‐animal PolX orthologs—particularly from microbial sources—remains largely untapped. In our previous work, semi‐rational engineering of *Bos taurus* TdT (BtTdT) yielded a variant (BtM5: Bt15AA^R336L/K338G/L397M/E456S/D395G^) that achieved complete substrate conversion and over 30‐fold catalytic enhancement [80]. Given the high structural conservation of the PolX core domain (PolXc) among TdTs and related PolX enzymes, we hypothesized that the five mutated residues in BtM5 might represent evolutionarily conserved hotspots for engineering other PolXs in order to enhance the activity toward both natural and unnatural dNTPs.

In this study, guided by structural and sequence analysis of PolX enzymes, we selected a *Faustovirus*‐derived polymerase (FvPolX) for enzyme engineering [81, 82]. Unlike AsfvPolX, FvPolX retains the full PolXc scaffold—including the 8 kDa, fingers, palm, and thumb subdomains. As a member of the NT_Pol *β*‐like superfamily, FvPolX shares greater sequence homology with Pol *β* than with other DNA polymerases. Through targeted mutagenesis of three key residues, we generated the FvPolX^R184L/T186G/N267S^ variant, which exhibited markedly improved catalytic efficiency toward both canonical and noncanonical 3′‐ONH_2_‐dNTPs. This variant enabled a functional transition from narrow to broad substrate recognition and from low to high catalytic activity. Further sequence truncation and site‐directed mutagenesis produced a truncated double mutant (s149FvPolX^R184L/N267S^) with enhanced catalytic activity. The retaining of only the palm and thumb subdomains suggested essential structural requirements for PolX functionality. Compared to wild‐type FvPolX, the s149FvPolX^R184L/N267S^ mutant demonstrated substantially enhanced catalytic activity toward all four natural dNTPs and 3′‐ONH_2_‐dNTPs, while completely eliminating the formation of −1 nt byproducts—similar to the full‐length triple mutant. Notably, the truncated variant showed superior incorporation efficiency for dTTP and dGTP relative to FvPolX^R184L/T186G/N267S^. This rational engineering approach for *de novo* enzymatic DNA synthesis by leveraging a previously unexplored viral PolX not only expands the enzyme toolkit with a non‐animal‐derived polymerase exhibiting novel catalytic traits, but also provides mechanistic insights into PolX‐mediated catalysis.

## 2 Results

### 2.1 Structural and Sequence Analysis of PolXs

**FIGURE 1:**
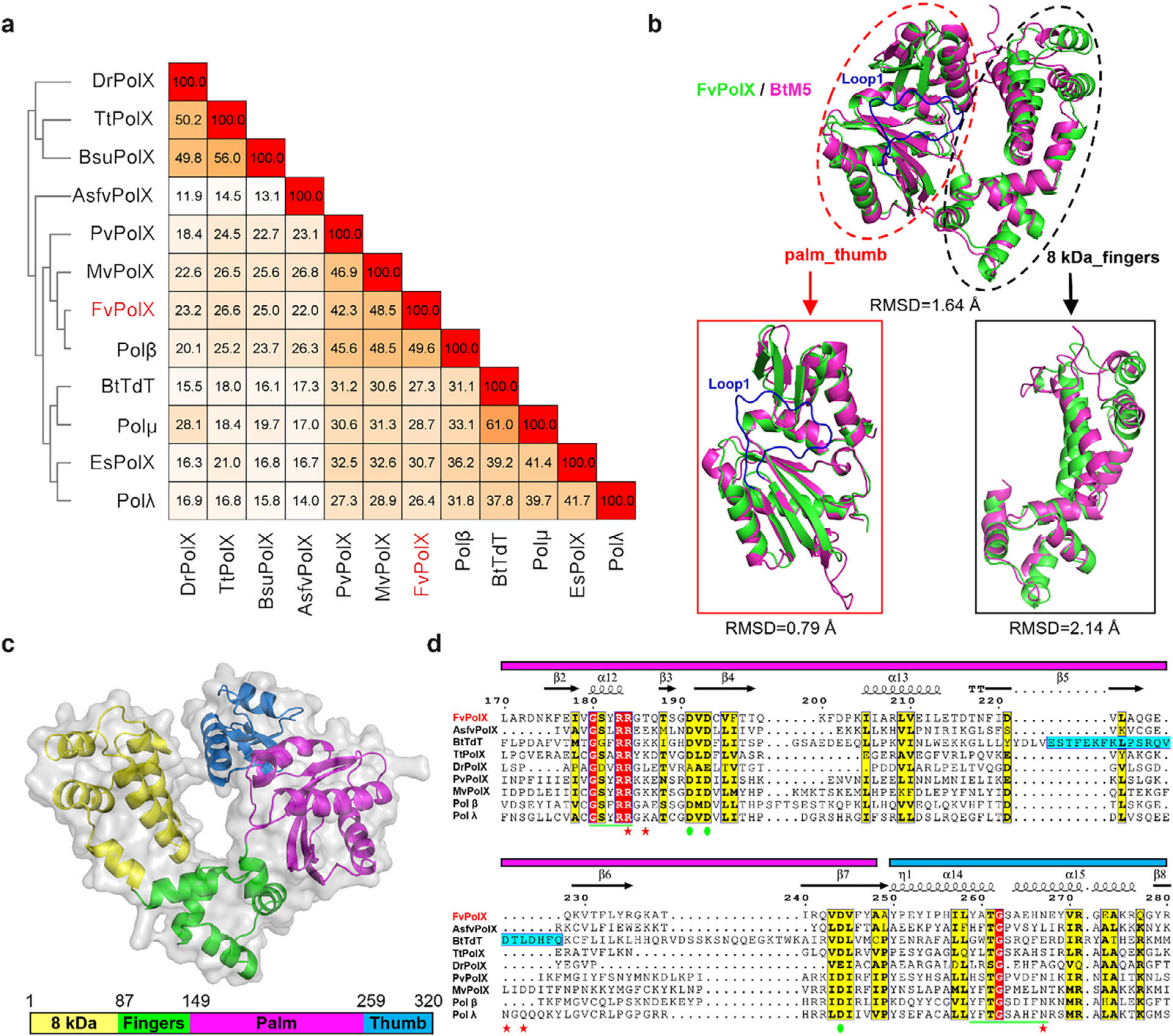
Structural and sequence analysis of FvPolX. (a) Phylogenetics analysis of PolX orthologs. Protein pairwise similarity matrix was generated using the TBtools‐II bioinformatics toolkit [84]. (b) Structural domain analysis of FvPolX. Superposition of the FvPolX structure (green, UniProt: A0A6M6DHZ3) and a modeled structure of BtM5 (i.e., Bt15AA^R336L/K338G/L397M/E456S/D395G^), an engineered variant of *Bos taurus* TdT (magenta, UniProt: P06526). Loop1 in BtM5 is shown in blue. (c) Predicted linear arrangement of the subdomains of FvPolX as predicted by AlphaFold3. The 8 kDa, fingers, palm, and thumb subdomains are colored in yellow, green, magenta, and blue, respectively. (d) Sequence alignment of the palm (magenta) and thumb (blue) domains in various PolX homologs. Catalytic triad residues (aspartate or glutamate) are marked with green circles; five key amino acid positions and the Loop1 region from the reference mutant BtM5 are indicated with red stars and a light blue background, respectively; two conserved motifs within the palm and thumb subdomains involved in dNTP binding are highlighted with light green lines; residues with a similarity score > 0.7 (based on the non‐redundant PolX dataset) are shown in bold, and absolutely conserved residues are represented in a red background. Species abbreviations: Tt, *Thermus thermophilus* (UniProt: Q5SJ64); Dr, *Deinococcus radiodurans* (UniProt: Q9RX48); pol *β*, pol *µ* and pol *λ*, human pol *β* (UniProt: P06746), pol *µ* (UniProt: Q9NP87) and pol *λ* (UniProt: Q9UGP5); Fv, *Faustovirus* (UniProt: A0A6M6DHZ3); Asfv, *African swine fever virus* (UniProt: P42494); Mv, *Mimivirus* (UniProt: A0A0G2Y8D8); Pv, *Pithovirus* (UniProt: A0A481Z4U2); Bsu, *Bacillus subtilis* (UniProt: P94544); Es, *Eutrema salsugineum* (NCBI: XP_024008683.1); Bt, *Bos taurus* (UniProt: P06526). Sequence alignments of PolXs were performed by Molecular Evolutionary Genetics Analysis using MEGA12 [85] software and visualized with the web server ESPript 3.0 [86].

Although PolXs are widely distributed in Animalia, Plantae, Fungi, Bacteria, Archaea, and Viruses, to the best of our knowledge, there has been no engineering work on any PolX from non‐animal sources for *de novo* DNA synthesis so far. In this study, thus, we sought to explore viral PolXs as a new resource for enzymatic DNA synthesis. To profile viral PolXs, we conducted a comprehensive bioinformatics analysis of diverse PolXs from the NCBI and UniProt databases (Tables S1 and S2). Sequence alignment showed that the highest identity/similarity to the reference BtTdT in animal‐derived Pol *µ* is 41.0%/61.0%, consistent with the established evolutionary origin of TdT as a specialized descendant of Pol *µ* [55], and followed by plant‐derived EsPolX (identity/similarity: 24.2%/39.2%) (Figure S1; Figure 1a). Viral PolXs containing an intact catalytic core domain (PolXc) exhibit approximately 30% similarity, whereas bacterial homologs show the lowest conservation (<20% similarity) with BtTdT. Phylogenetically related PolX enzymes from the same taxonomic origin exhibit pronounced sequence conservation, forming discrete evolutionary clusters. Bacterial representatives (e.g., *Thermus thermophilus* TtPolX, *Deinococcus radiodurans* DrPolX, and *Bacillus subtilis* BsuPolX) demonstrate intra‐group sequence identities exceeding 30% and similarities above 49%. In contrast, most viral representatives exhibit identities above 26% and similarities over 42% (with the exception of AsfvPolX). Phylogenetic analysis indicates that viral PolXs are evolutionarily closer to BtTdT than their bacterial counterparts, offering a promising template for enzyme engineering.

To further refine candidate selection, structural similarity was evaluated using RMSD as the primary evaluation metric to assess the core catalytic PolXc domain alignment. Structural comparisons identified Pol *µ* and Pol *λ* as the closest structural homologs to BtTdT (backbone RMSD <1.50 Å), followed by viral‐derived FvPolX (RMSD 1.64 Å) and AsfvPolX (RMSD 1.76 Å) (Figure S2). In contrast, bacterial PolXs show significantly lower structural similarity, with consensus RMSD values exceeding 8 Å (Figure S2). Among viral candidates, FvPolX displays the highest structural resemblance to the PolXc structure of BtTdT and was therefore selected for further engineering. Conserved domain analysis using NCBI Conserved Domain Database confirmed that FvPolX belongs to the NT_PolXc and NT_Pol *β*‐like superfamilies and retains essential catalytic motifs, including the metal‐binding triad, nucleotide‐binding sites, active site residues, and primer‐binding motifs [83] (Figure S3).

We further analyzed subdomain‐level structural differences between FvPolX and the PolXc structure of BtTdT. It was found that FvPolX retains the complete conserved PolXc catalytic core architecture, comprising the 8 kDa, fingers, palm, and thumb subdomains (Figure 1c). Among these, the palm and thumb subdomains—primarily involved in catalysis and DNA binding—exhibit high structural conservation with BtTdT (RMSD = 0.79 Å). In contrast, the 8 kDa and fingers subdomains—responsible for DNA and nucleotide substrate binding—show greater divergence (RMSD = 2.14 Å) (Figure 1b,c). A notable structural distinction was observed in the palm subdomain: BtTdT contains a longer Loop1 (21 residues, Figure 1b), whereas FvPolX displays a deletion of Loop1 with a distinct backbone topology (Figure 1b,d). Given the high structural similarity between FvPolX and BtTdT, along with the rationally modified BtTdT mutant BtM5 with five point mutations that significantly enhanced catalytic activity, we speculated that these five residues might also be critical amino acids in FvPolX. Corresponding modifications at these sites in FvPolX were expected to improve its catalytic activity toward mononucleotide substrates. Therefore, we proceeded to generate mutations at the corresponding five sites in FvPolX. Due to the absence of a canonical Loop1 in FvPolX, two corresponding amino acid residues present in the variant BtM5 are missing, limiting the number of reference positions available for mutation to three: R184, T186, and N267. Of note, FvPolX is classified as a canonical PolX enzyme, characterized by a conserved catalytic triad of three acidic residues that coordinate catalytically essential metal ions, likely conferring intrinsic DNA polymerase activity (Figure 1d).

### 2.2 Catalytic Profiling and Active‐Site Remodeling of FvPolX by Rational Mutagenesis

**FIGURE 2:**
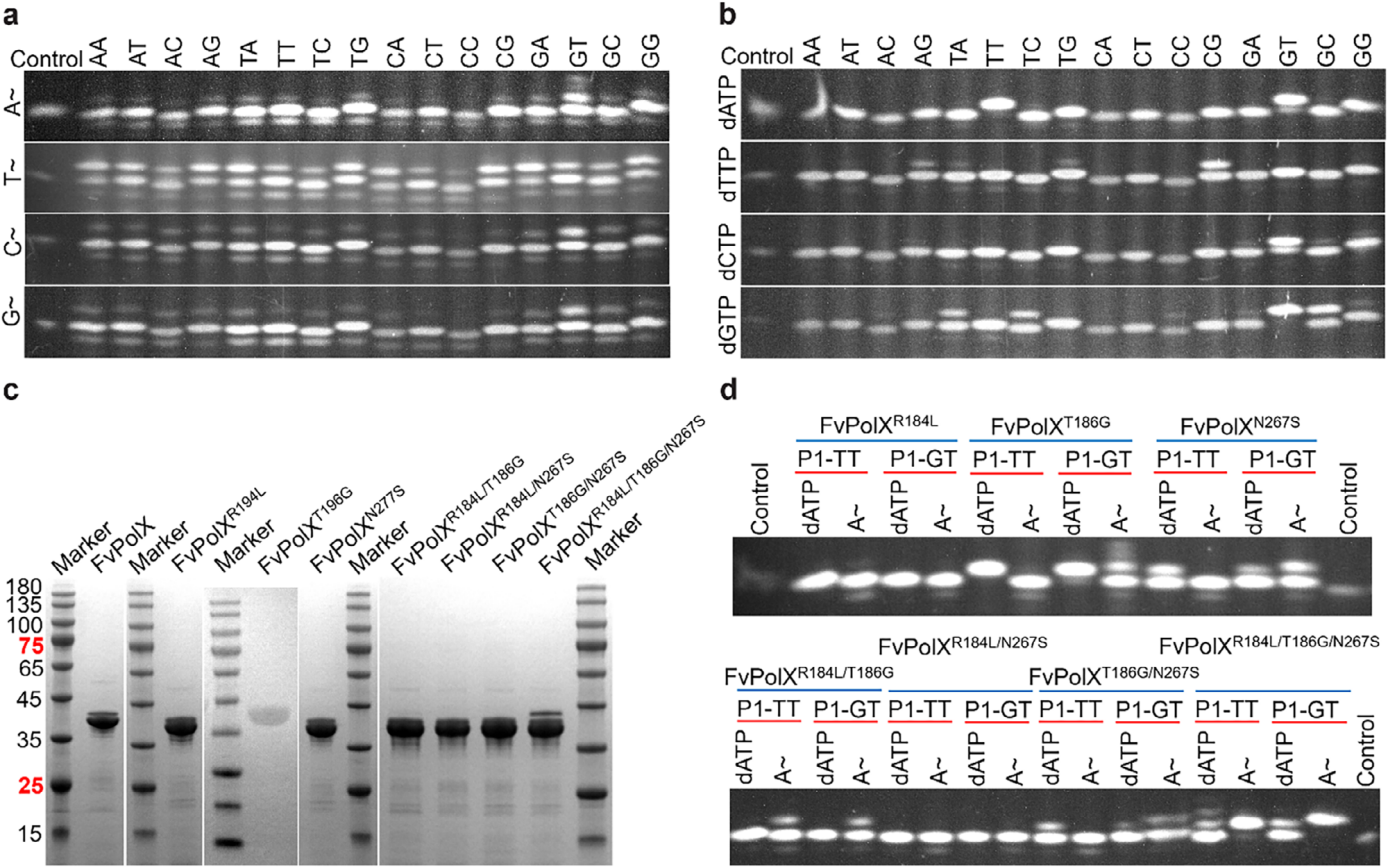
Analysis of the catalytic activities of FvPolX and its mutants. (a) Extension of four types of unnatural substrates 3′‐ONH_2_‐dNTPs across 16 iDNA substrates with varied terminal dinucleotide sequences by wild‐type FvPolX. (b) Extension of natural substrates dNTPs across 16 iDNA substrates by wild‐type FvPolX. (c) SDS‐PAGE analysis of purified recombinant FvPolX mutants. (d) Extension activities of FvPolX single, double, and triple mutants using either dATP or 3′‐ONH_2_‐dATP as substrates. Reaction conditions: 1 mg/mL FvPolX or its mutants, 1 µM iDNA, 30°C, 3 min for dATP and 25 min for 3′‐ONH_2_‐dATP. “A∼”, “T∼”, “C∼”, and “G∼” stand for 3′‐ONH_2_‐dATP, 3′‐ONH_2_‐dTTP, 3′‐ONH_2_‐dCTP, and 3′‐ONH_2_‐dGTP, respectively.

Wild‐type FvPolX (*N*‐terminally fused to a hexa‐histidine tag, His_6_, for protein purification) was heterologously expressed and purified from *Escherichia coli* JM109 (DE3) using nickel‐affinity chromatography. Prior to engineering, we evaluated the single‐stranded DNA extension activity of FvPolX using a panel of 16 initiator DNA (iDNA) substrates with varying terminal dinucleotide pairs, in combination with either natural dNTPs or unnatural 3′‐ONH_2_‐dNTPs (Figure 2a,b). The 3′‐ONH_2_‐dNTPs contain a reversible ONH_2_ blocking group that can be chemically removed by nitrous acid to regenerate the native 3′‐OH group, and have been successfully employed in *de novo* DNA synthesis for both research and industrial applications [7, 9, 14, 87, 88, 89]. For reactions using 3′‐ONH_2_‐dNTPs, all 64 substrate combinations produced −1 frameshift products (i.e., −1 nt byproducts). Gel electrophoresis results were quantitatively analyzed using ImageJ software by calculating standardized densitometry values of the corresponding bands across all gels [90]. Among the tested combinations, the GA + T∼ substrate gave the highest conversion efficiency (≈70%), while no detectable +1 nt extension products were observed for CC + A∼, CC + G∼, and CT + A∼. In contrast, for natural dNTPs substrates, FvPolX displayed narrow substrate scope, catalyzing only 17 out of 64 substrate combinations under the tested conditions (TT/GT + dATP, AG/TA/TG/CG + dTTP, GA/GT/GC/GG + dCTP, and TA/TC/CC/GA/GT/GC/GG + dGTP). Among these, only three combinations achieved >90% conversion efficiency (TT/GT + dATP, and GT + dGTP). The −1 frameshift products in the case of iDNA substrates extended by FvPolX might arise from its dismutase activity, similar to that of TdT [91], meaning that FvPolX is able to cleave a base off from the 3′‐end of one iDNA substrate. Overall, wild‐type FvPolX exhibited significantly lower catalytic efficiency toward both natural and non‐natural dNTPs substrates when compared to previously reported wild‐type TdTs [4, 6, 25], suggesting that engineering a high‐activity variant of FvPolX poses a greater challenge than for previously optimized polymerases such as BtTdT. In addition to cobalt (II) ions, PolXs were reportedly able to utilize other metal ions for catalysis [8, 50, 63]. Thus, we tested the effects of different metal ions (monovalent and divalent ions) on its enzymatic activity. As a result, FvPolX showed varying activities with Co^2+^, Cu^2+^, Fe^2+^, Mg^2+^, Mn^2+^, or Zn^2+^ as the metal cofactor, exhibiting the highest elongation activity in the presence of Co^2+^ or Zn^2+^ (Figure S4).

Building on our previous rational redesign of BtM5, which eliminated single‐nucleotide deletion byproducts and achieved over 30‐fold catalytic enhancement by targeting five critical residues, we investigated whether similar modifications could also enhance FvPolX activity, particularly toward non‐natural 3′‐ONH_2_‐dNTPs substrates. Despite moderate sequence identity (27.3%, Figure 1a), FvPolX and BtTdT share high structural conservation within the PolXc domain (RMSD = 1.64 Å; Figure 1b), enabling identification of equivalent positions for mutagenesis. Notably, FvPolX lacks two residues corresponding to BtTdT's Loop1 region, leaving only three accessible sites (R184, T186, and N267) for targeted mutagenesis. Guided by mechanistic insights gained from BtM5 polymerases, we prepared three single mutants (R184L, T186G, and N267S), three double mutants (R184L/T186G, R184L/N267S, and T186G/N267S), and one triple mutant (R184L/T186G/N267S) (Figure 2c). All mutant variants were expressed as soluble proteins, with each yielding approximately 10 mg of each purified target protein per liter of LB culture following affinity purification. Catalytic activities of these mutants were then quantitatively assessed using both canonical dATP and the 3ʹ‐amino‐blocked analog 3′‐ONH_2_‐dATP (Figure 2d). Among the single‐point mutants, only FvPolX^T186G^ retained activity comparable to the wild type enzyme, while FvPolX^R184L^ and FvPolX^N267S^ displayed decreased catalytic efficiency—particularly FvPolX^R184L^, which lost all activity toward dATP. Most notably, the triple mutant achieved suppression of −1 nt frameshift errors and substantially enhanced incorporation of 3′‐ONH_2_‐dATP, achieving 100% conversion for the GT + 3′‐ONH_2_‐dATP substrate, compared to < 50% conversion for the wild‐type enzyme (Figure 2d). Collectively, mutational engineering at these three sites modulates FvPolX's catalytic activity across three distinct facets: 1) suppression of −1 nt byproduct formation; 2) reprogramming substrate selectivity in favor of 3′‐modified dATP analogues, and 3) exhibition of the TA + dATP activity (visible as the top band in the TT + dATP lane).

### 2.3 Catalytic Profiling of FvPolX^T186G^ and FvPolX^R184L/T186G/N267S^

**FIGURE 3:**
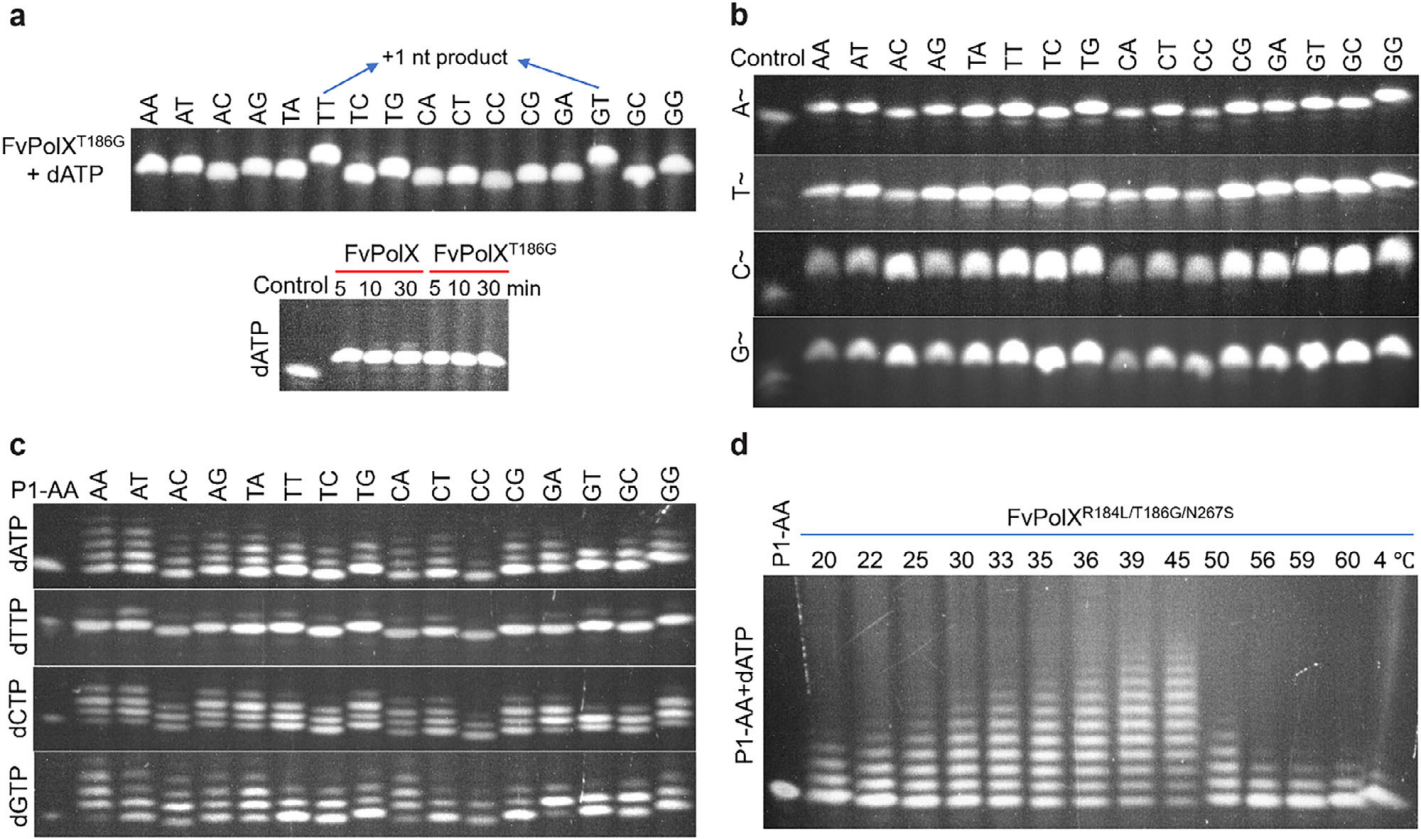
Analysis of the catalytic activities of FvPolX mutants. (a) Elongation of dATP by the FvPolX^T186G^ single mutant and time‐course analysis for iDNA P1‐TT. (b) Elongation of four types of unnatural 3′‐ONH_2_‐dNTPs with 16 iDNA substrates bearing varied terminal dinucleotide sequences by FvPolX^R184L/T186G/N267S^. (c) Elongation of four types of natural substrates dNTPs with 16 iDNA substrates by FvPolX^R184L/T186G/N267S^. (d) Optimal reaction temperature of FvPolX^R184L/T186G/N267S^. Reaction conditions: 1 mg/mL FvPolX mutants, 1 µM iDNA, 30°C for 3 min with natural dNTPs and 25 min with 3′‐ONH_2_‐dNTPs. “A∼”, “T∼”, “C∼”, and “G∼” stand for 3′‐ONH_2_‐dATP, 3′‐ONH_2_‐dTTP, 3′‐ONH_2_‐dCTP, and 3′‐ONH_2_‐dGTP, respectively. For the optimal‐temperature assay, 0.45 mg/mL FvPolX^R184L/T186G/N267S^ was used with 1 µM of P1‐AA (Table S3) primer for 10 min.

The FvPolX^T186G^ mutant enabled controllable single‐nucleotide extension at TT/GT termini using dATP, yielding exclusively +1 nt products. Even after prolonged incubation following complete conversion to the +1 nt species, no detectable +2 nt products were observed (Figure 3a). The dGTP, dCTP, or dTTP incorporation activities of the FvPolX^T186G^ protein were also tested (Figure S5). We found that FvPolX^T186G^ was unable to catalyze the polymerization of P1‐TT+ dT/C/GTP and of P1‐GT + dTTP.

Building on the preliminary results showing that the triple mutant FvPolX^R184L/T186G/N267S^ enhanced the catalytic efficiency toward both TT/GT + 3′‐ONH_2_‐dATP and TT/GT + dATP substrates, we systematically profiled its activity across all 16 iDNAs (differing in terminal dinucleotides), with each extended by four types of 3′‐ONH_2_‐dATP (Figure 3b) and four types of natural dATP (Figure 3c) combinations. Notably, this mutant exhibited broad and robust activities toward 3′‐ONH_2_‐dNTPs: complete conversions were observed for 16 iDNAs and 3′‐ONH_2_‐dC/GTP combinations, in sharp contrast to the wild‐type FvPolX, which showed <50% yield and a −1 nt byproduct (Figure 2a). Reduced efficiency was selectively observed when incorporating 3′‐ONH_2_‐dATP at TT/TC/TG termini or 3′‐ONH_2_‐dTTP at TA/TT/TG termini (unconverted substrate <6%, Figure 3b), suggesting that steric hindrance impedes elongation at iDNAs containing a penultimate T. The FvPolX^R184L/T186G/N267S^ mutant also demonstrated broadly enhanced activities toward natural dNTPs, catalyzing processive extension products of +1 to +4 nt across most iDNA‐dNTPs combinations. However, dTTP incorporation efficiency remained comparable to that of the wild‐type enzyme, indicating steric constraints persist at thymidine insertion sites. Interestingly, this mutant displayed attenuated activity in four substrate combinations compared to the wild‐type enzyme, including GT + dA/C/GTP and CG + dTTP, with complete loss of activity observed specifically for CG + dTTP incorporation.

Leveraging the improved catalytic performance toward dNTPs, we assessed the temperature optimum of FvPolX^R184L/T186G/N267S^ using the AA + dATP substrate pair. Activity profiling revealed the peak performance at 45°C. And FvPolX^R184L/T186G/N267S^ experienced a sharp decline in activity at temperatures ≥50°C, with over 70% reduction in product yield (Figure 3d). Additionally, we compared the catalytic activity of FvPolX^R184L/T186G/N267S^ toward 3′‐ONH_2_‐dNTPs with two previously reported wild‐type TdTs (BtTdT and ZaTdT) and a select number of mutants (ZaTdT‐R335L‐K337G, ZaTdT‐R335L‐A193T‐G337H‐H478G, BtM5, and M7−8/LG) at reaction temperatures of 30°C and 45°C (Figure S6 and Table S2) [6, 9, 40]. Under the 45°C reaction conditions, the activities of these tested enzymes in P1‐CC extending with 3′‐ONH_2_‐dATP increased in the following order: BtTdT < ZaTdT < ZaTdT‐R335L‐K337G < ZaTdT‐R335L‐A193T‐G337H‐H478G < FvPolX^R184L/T186G/N267S^ ≈ BtM5< M7−8/LG. Under the 30°C reaction conditions, ZaTdT‐R335L‐A193T‐G337H‐H478G and FvPolX^R184L/T186G/N267S^ exhibited a similar activity level, and their conversion rates were higher than those observed at 45°C. Comparatively, FvPolX^R184L/T186G/N267S^ demonstrated better thermal stability than ZaTdT‐R335L‐A193T‐G337H‐H478G. To better demonstrate FvPolX^R184L/T186G/N267S^’s potential, especially in *de novo* DNA synthesis, we attempted to synthesize 4‐nt DNA (ATCG) by FvPolX^R184L/T186G/N267S^ using 5′‐biotin modified iDNA P1‐AA (biotin‐P1‐AA), which was preimmobilized onto streptavidin modified magnetic beads via biotin‐streptavidin chemistry, and the products were purified via magnetic separation at each step. To our delight, we successfully synthesized the 4‐nt final product (Figure S7). However, the yield was low, indicating that the synthetic process needs to be optimized in the future.

### 2.4 Truncation of FvPolX Mutants

**FIGURE 4:**
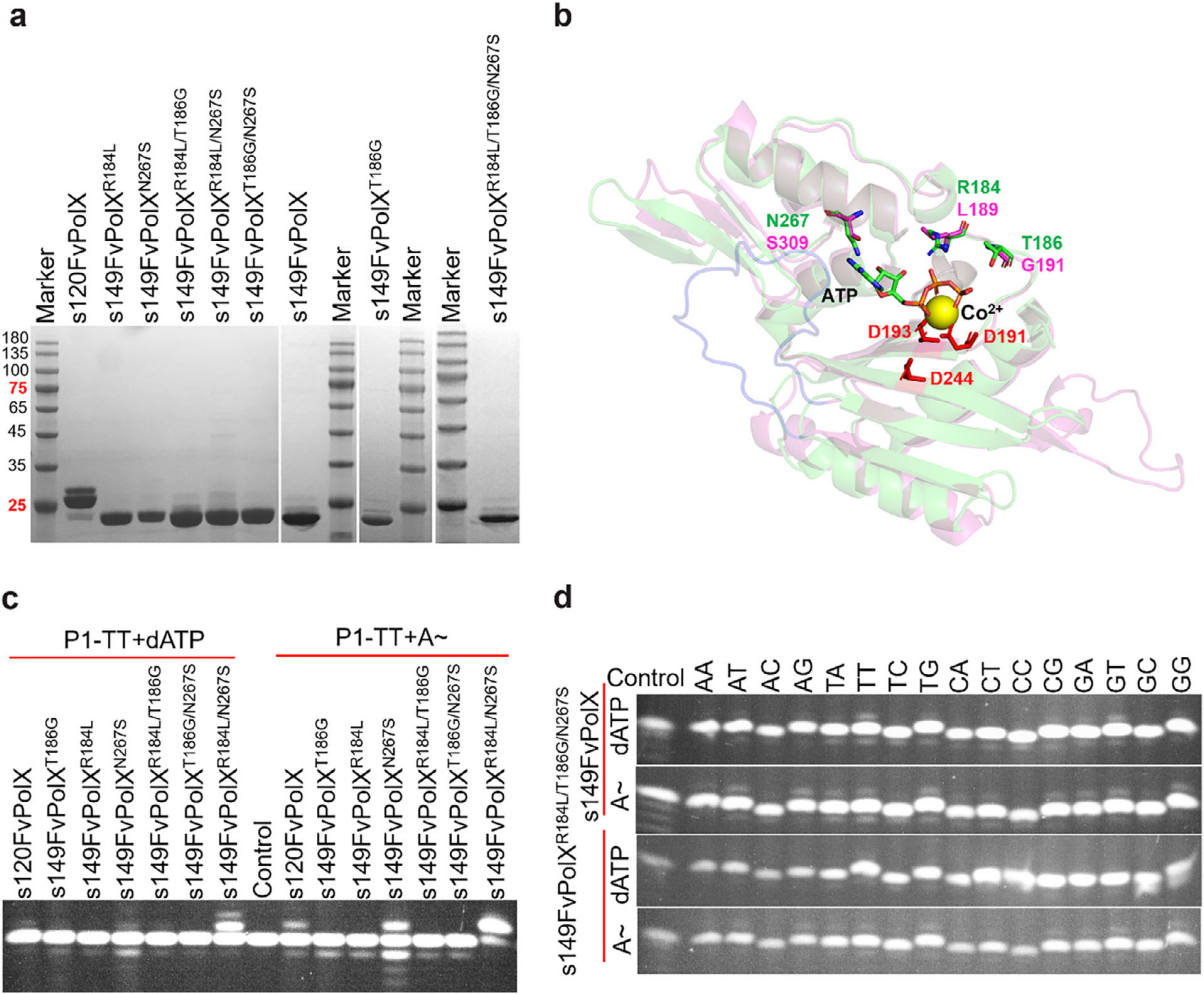
Analysis of the catalytic activities of truncated FvPolX mutants. (a) SDS‐PAGE analysis of purified truncation mutants of FvPolX. (b) Structural comparison of three mutant amino acid residues in truncated FvPolX (green) and BtM5 (magenta), with the incoming dATP shown in stick representation. The catalytic triad residues (aspartates) in s149FvPolX are depicted as red sticks. (c) Elongation activities of truncated FvPolX mutants with dATP and 3′‐ONH_2_‐dATP. (d) Elongation activities of truncated s149FvPolX and s149FvPolX^R184L/T186G/N267S^ for dATP and 3′‐ONH_2_‐dATP. Reaction conditions: 1 mg/mL truncated FvPolX mutants, 1 µM iDNA, 30°C, 25 min for both dATP and 3′‐ONH_2_‐dATP. “A∼” stands for 3′‐ONH_2_‐dATP. The controls in (c,d) refer to P1‐TT and P1‐AA, respectively.

Inspired by AsfvPolX—a minimal PolX‐family polymerase that retains physiological activity with only the catalytic palm and dNTP/DNA‐binding thumb subdomains—we engineered a number of *N*‐terminally truncated FvPolX variants preserving solely these two domains (Figure 4a,b). These subdomains alone were sufficient to catalyze template‐independent DNA polymerization without the accessory domains present in the full‐length enzyme. The catalytic triad residues (D191, D193, D244) are proposed to coordinate a cobalt (II) ion, which facilitates nucleotide binding and positioning for efficient phosphodiester bond formation (Figure 4b). Subsequently, we examined the activities of truncated variants and assessed whether substitutions at the three key residues also conferred catalytic improvement in these constructs. As a result, two truncated constructs, s120FvPolX (residues 120–320, Table S2) and s149FvPolX (residues 149–320), displayed weak activity for TT + 3′‐ONH_2_‐dATP (<9% yield) and markedly reduced activity for TT + dATP (<6% yield) when compared with the wild‐type enzyme (∼100% yield) (Figure 4c,d).

The truncated variant s149FvPolX (∼19.8 kDa) represents the smallest functional polymerase scaffold. We therefore implemented structure‐guided mutagenesis to enhance its catalytic efficiency. Combinatorial point mutations (single/double/triple) were subsequently introduced into the s149FvPolX truncation backbone. These engineered variants exhibited improved soluble expression, yielding ∼13 mg of each purified protein per liter of LB culture following affinity chromatography (Figure 4a). Catalytic activities against both 3′‐ONH_2_‐dATP and dATP were then evaluated using TT‐terminated iDNA. Among the truncated mutants screened, only 149FvPolX^R184L/N267S^ showed substantial enhancement (>6‐fold relative to s149FvPolX‐WT) with the TT/dATP substrate pair, producing +1/+2 nt extension products (Figure 4c). Other single and double mutants only exhibited a negligible activity. Notably, s149FvPolX^R184L/N267S^ also showed markedly improved activity with the TT/A∼ substrate pair (∼84.3% yield) when compared with s149FvPolX‐WT and s120FvPolX‐WT (>10‐fold increase) and full‐length WT (>35‐fold increase), eliminating −1 nt aberrant products. The FvPolX^R184L/N267S^ variant exhibited undetectable catalytic activity toward TT+A∼. In contrast, the full‐length triple mutant FvPolX^R184L/T186G/N267S^ exhibited strong enhancement toward most substrate pairs, whereas the truncated triple mutant s149FvPolX^R184L/T186G/N267S^ showed negligible catalytic activity (Figure 4d).

**FIGURE 5:**
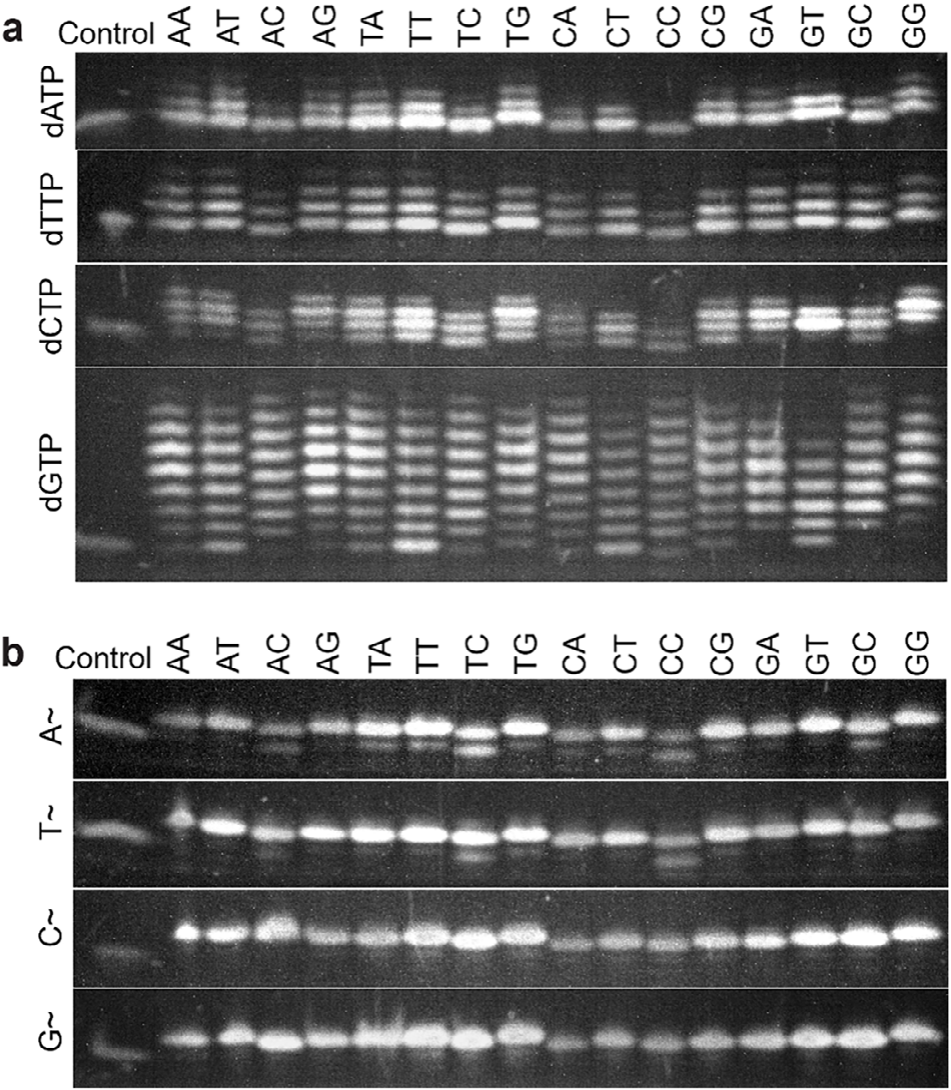
Analysis of the catalytic activities of the truncation mutant s149FvPolX^R184L/N267S^. (a) Elongation activity with natural dNTPs substrates for 16 iDNAs. (b) Elongation activity with 3′‐ONH_2_‐dNTPs substrates for 16 iDNAs. Reaction conditions: 1 mg/mL s149FvPolX^R184L/N267S^, 1 µM iDNA, 30°C, 3 min for dATP and 25 min for 3′‐ONH_2_‐dATP. “A∼”, “T∼”, “C∼”, and “G∼” stand for 3′‐ONH_2_‐dATP, 3′‐ONH_2_‐dTTP, 3′‐ONH_2_‐dCTP, and 3′‐ONH_2_‐dGTP, respectively.

Given the enhanced activity of s149FvPolX^R184L/N267S^ with TT + 3′‐ONH_2_‐dATP relative to both full‐length and truncated controls (s120FvPolX and s149FvPolX) (Figures 2a and 4c,d), we further examined its catalytic performance with natural dNTPs and 3′‐ONH_2_‐dNTPs analogs across 16 iDNA substrates (Figure 5a,b). Unlike FvPolX^R184L/T186G/N267S^—which improved the activity for only three natural dNTPs (excluding dTTP)—s149FvPolX^R184L/N267S^ exhibited pronounced enhancement across all four natural dNTPs, with the most dramatic effect for dGTP. In particular, while wild‐type FvPolX showed no detectable activity toward AA/AT + dGTP, both FvPolX^R184L/T186G/N267S^ and s149FvPolX^R184L/N267S^ restored measurable activity, with the latter outperforming the former by 2.7/3.4‐fold under the testing conditions (Figures 2b, 3c, and 5a). s149FvPolX^R184L/N267S^ generated discrete extension products ranging from +1 to +9 nt, whereas FvPolX^R184L/T186G/N267S^ yielded +1 to +4 nt products. Consistent with FvPolX^R184L/T186G/N267S^, s149FvPolX^R184L/N267S^ displayed predominantly processive polymerization during dNTPs incorporation. Furthermore, s149FvPolX^R184L/N267S^ showed strong enhancement toward all four 3′‐ONH_2_‐dNTPs, with concomitant elimination of −1 nt deletion byproducts (Figure 5b). The truncation mutant s149FvPolX^R184L/N267S^ achieved near‐complete conversion with 3′‐ONH_2_‐dC/GTP substrates, while maintaining partial conversion (>50%) for 3′‐ONH_2_‐dA/TTP substrates. Substrates such as TC/CC+3′‐ONH_2_‐dATP and CC+3′‐ONH_2_‐dTTP showed the lowest efficiencies, with conversion ratios of 69.5%, 50.6%, and 64.9%, respectively. Truncation mutagenesis of FvPolX polymerase revealed that retention of merely two core structural subdomains (palm and thumb) suffices for proficient template‐independent DNA polymerase activity. While s149FvPolX^R184L/N267S^ did not represent the optimal variant, its catalytic efficiency could be enhanced through further protein engineering to meet practical application requirements, which is ongoing in this laboratory.

## 3 Conclusions

Given the broad distribution of PolX family enzymes across phylogenetic lineages and their conserved structure‐function architecture, we sought to expanding research beyond animal‐derived homologs. Leveraging our previous engineering of BtTdT [80], we demonstrate that key amino acid residues possess transferable functional roles in PolX enzyme engineering. Structurally, FvPolX lacks the longer Loop1 domain present in BtTdT, which leads to the absence of two catalytically critical residues in the Loop1 domain of BtTdT. Introducing the remaining three BtM5 residues into full‐length or truncated FvPolX variants broadened the substrate scope to include both dNTPs and 3′‐NH_2_‐dNTPs, while markedly enhancing catalytic activity and eliminating −1 nt byproduct formation. The s149FvPolX^R184L/N267S^ truncation mutant (residues 149–320) exhibited substantially enhanced catalytic activity compared with full‐length and truncated FvPolX. This variant also outperformed the FvPolX^R184L/T186G/N267S^ triple mutant in dT/GTP incorporation. The mechanistic basis for these improvements remains unclear; future structural and kinetic analyses will clarify the underlying principles and guide the rational engineering of next‐generation variants. Given that the s149FvPolX^R184L/N267S^ mutant retains only about half the protein sequence of full‐length FvPolX, this minimized scaffold facilitates more efficient screening and rational design.

In summary, our engineering of FvPolX for enzymatic *de novo* DNA synthesis expands both the species diversity and scaffold landscape of DNA polymerases, providing new biocatalytic tools for this rapidly progressing field. We further demonstrate the conserved regulatory role of three catalytic triad residues (R184, T186, N267) across the PolX family, providing a broadly applicable framework for polymerase optimization. Engineered FvPolX mutants and other non‐TdT PolX variants can serve as strategic alternatives to TdT in specialized applications, thereby broadening the enzymatic toolbox for tailored DNA synthesis.

## 4 Experimental Section/Methods

### 4.1 Strains, Media, and Chemicals

*E. coli* DH5α (Shanghai Weidi Biotechnology, China) was used for plasmid construction and cloning, while *E. coli* JM109 (DE3) (Shanghai Weidi Biotechnology, China) was used for enzyme expression. Luria‐Bertani (LB) medium (0.5% yeast extract, 1% tryptone, and 1% NaCl) was used to cultivate *E. coli* strains for plasmid amplification and extraction, seed preparation, and protein expression and purification. Ampicillin (60 mg/L) was added to the medium to maintain plasmids, and 1 mm isopropyl *β*‐D‐1‐thiogalactopyranoside (IPTG) was added to induce target protein expression. SYBR Gold nucleic acid gel stain was purchased from Thermo Fisher Scientific (USA) and 3′‐ONH_2_‐dNTPs from Firebird Biomolecular Sciences (Florida, USA).

### 4.2 Construction of Plasmids and Strains

All FvPolX wild‐type and mutant enzymes (Table S2) were codon‐optimized for *E. coli* expression and synthesized by BGI Tech Solutions (Beijing Liuhe) (Beijing, China). The primers used in this study are listed in Table S3. The FvPolX genes were amplified by PCR using PrimeSTAR Max DNA Polymerase (Takara Biomedical Technology (Beijing, China)). Plasmids were constructed via homologous recombination using the ClonExpress Ultra One Step Cloning Kit purchased from Nanjing Vazyme Biotech (Nanjing, China). FvPolX genes were inserted into the pETDuet‐1 plasmid to express proteins carrying an *N*‐terminal His_6_‐tag. Vector pETDuet‐1 was digested with restriction enzymes *Bam*HI *and Not*I. The recombinant plasmids were first transformed into cloning strains, and single colonies were picked and sent to Sangon Biotech (Qingdao, China) for DNA sequencing. The verified recombinant plasmids were then transformed into *E. coli* JM109 (DE3) for protein expression and purification. Details of the corresponding processes are provided in Table S3.

### 4.3 Protein Expression and Purification

*E. coli* JM109 (DE3) harboring the relevant recombinant vector was grown overnight in 10 mL LB medium containing 100 µg/mL ampicillin at 37°C with shaking at 200 rpm. The cultures were used as seeds to inoculate 1 L LB medium containing the same antibiotic and grown at 37°C and 200 rpm. When the culture optical density (OD_600_) reached approximately 0.7, the culture temperature was lowered to 20°C, and IPTG was added to a final concentration of 0.8 mm to induce protein expression at 200 rpm for 20 h. Cells were harvested by centrifugation (6000 ×*g*, 7 min, 4°C), and cell pellets were resuspended in 60 mL precooled lysis buffer (50 mm Tris‐HCl, glycerol 10%, pH 7.5). All purification steps were performed at 4°C. First, an Ultrasonic Homogenizer (JY92‐IIDN) (Ningbo Scientz Biotechnology, China) was used for ultrasonication‐based cell lysis (2 s on, 4 s off, and 25 min for total working time). After centrifugation at 9000 ×*g* for 50 min to remove cell debris, the supernatant was loaded onto a Ni‐NTA agarose column (Qiagen, Germany). The column was washed eight times with 10 mL lysis buffer containing 50 mm imidazole, and the target proteins were eluted with protein elution buffer (50 mm Tris‐HCl, glycerol 10%, 250 mm imidazole, pH 7.5). An Amicon Ultra centrifugal filter with a 30 KDa cutoff (Merck KGaA, Germany) was used to concentrate the eluted proteins at 5000 ×*g* for 25 min, and the elution buffer was exchanged three times with desalting buffer (100 mm Tris‐HCl, 40% glycerol, pH 7.5). Protein concentrations were determined by a NanoDrop One^C^ spectrophotometer (Thermo Fisher Scientific, USA). Finally, protein aliquots (50 µL per tube) were snap‐frozen in liquid nitrogen and stored at −80°C until use.

### 4.4 Accurate Activity Measurement Based on Fluorescent Dye and Gray Value Calculations

The FvPolX enzymatic reaction system for natural dNTPs/3′‐ONH_2_‐dNTPs contained 1 mg/mL purified FvPolX, 1 µM iDNAs (Table S3), 1 mm each of the four kinds of dNTPs or 3′‐ONH_2_‐dNTPs, 1 mm CoCl_2_, and DNA synthesis buffer (15% (*v*/*v*) DMSO, 50 mm *O*‐benzylhydroxylamine·HCl, 10% (*v*/*v*) glycerol, 0.05% (*v*/*v*) Tween 20, 2.5 mm tris‐HCl, 40 mm NaCl, 0.5 mm Hepes, 0.5 m cacodylic acid, and a final pH of 7.0 (measured in the absence of DMSO)). Reactions incubated at 30°C for 3 min (dNTPs) or 25 min (3′‐ONH_2_‐dNTPs) and then heated to 75°C for 6 min to quench the reaction. The DNA products were analyzed on a 20% polyacrylamide gel with 8 m Urea electrophoresis (DNA Urea PAGE), using a DNA Urea PAGE Electrophoresis Kit purchased from Real‐Times (Beijing) Biotechnology (Beijing, China). Gels were stained with SYBR Gold nucleic acid gel dye and imaged with a Tanon 1600 Gel Image System (Tanon, China), and the (+ n) products were quantified using ImageJ [90] software based on gray value calculations.

### 4.5 Determination of the Optimal Enzyme Reaction Temperature of FvPolX^R184L/T186G/N267S^

The standard reaction system consisted of 0.45 mg/mL purified FvPolX^R184L/T186G/N267S^, 1 µm iDNAs (P1‐AA; Table S3), 1 mm dATP, 1 mm CoCl_2_, and 100 mm Tris‐HCl buffer (pH 7.0). Samples were incubated at different temperatures from 4°C to 60°C for 10 min and then heated to 75°C for 6 min to quench the reaction. For optimal temperature determination, reaction mixtures were aliquoted into separate tubes and incubated at respective reaction temperatures for 10 min.

## Author Contributions

S.L., L.D., and C.Z. designed the study and analyzed the data. C.Z. performed the experiments. C.Z., L.D., and S.L. wrote the manuscript.

## Funding

This work was supported by the National Key Research and Development Program of China (2025YFA0923200, 2025YFD1700400), the National Natural Science Foundation of China (32370032), the Shandong Provincial Natural Science Foundation (ZR2020ZD23), the Taishan Young Scholars (tsqn202312032), and The Fundamental Research Funds of Shandong University (2023QNTD001). [Correction added on 29 May 2026, after first online publication: grant number of National Key Research and Development Program of China has been updated in this version.]

## Conflicts of Interest

The authors declare no conflicts of interest.

## Supporting information




**Supporting File**: advs75017‐sup‐0001‐SuppMat.pdf.

## Data Availability

All data are available in the main text or the supplementary materials.
