# Supplementary material for: Engineering a Virus‐Derived X Family DNA Polymerase FvPolX for de novo DNA Synthesis
Source: Adv Sci (Weinh). 2026 Mar 31;13(32):e75017. doi: 10.1002/advs.75017 (PMC13252658; doi:10.1002/advs.75017)
Supplement: Supplementary file 1 — Supporting File: advs75017‐sup‐0001‐SuppMat.pdf. [file ADVS-13-e75017-s001.pdf]

Engineering a Virus-derived X Family DNA Polymerase FvPolX for *de novo* DNA Synthesis

Chengjie Zhang[1]*, Lei Du*[1,]**, and Shengying Li*[1,2,]***

C. Zhang, L. Du and S. Li

[1]State Key Laboratory of Microbial Technology, Shandong University, Qingdao, Shandong, 266237, China

E-mail: lei.du@sdu.edu.cn (L.D.)

lishengying@sdu.edu.cn (S.L.)

S. Li

[2]Laboratory for Marine Biology and Biotechnology, Qingdao Marine Science and Technology Center, Qingdao, Shandong, 266237, China

The PDF file includes:
Figures S1 to S7
Tables S1 to S3

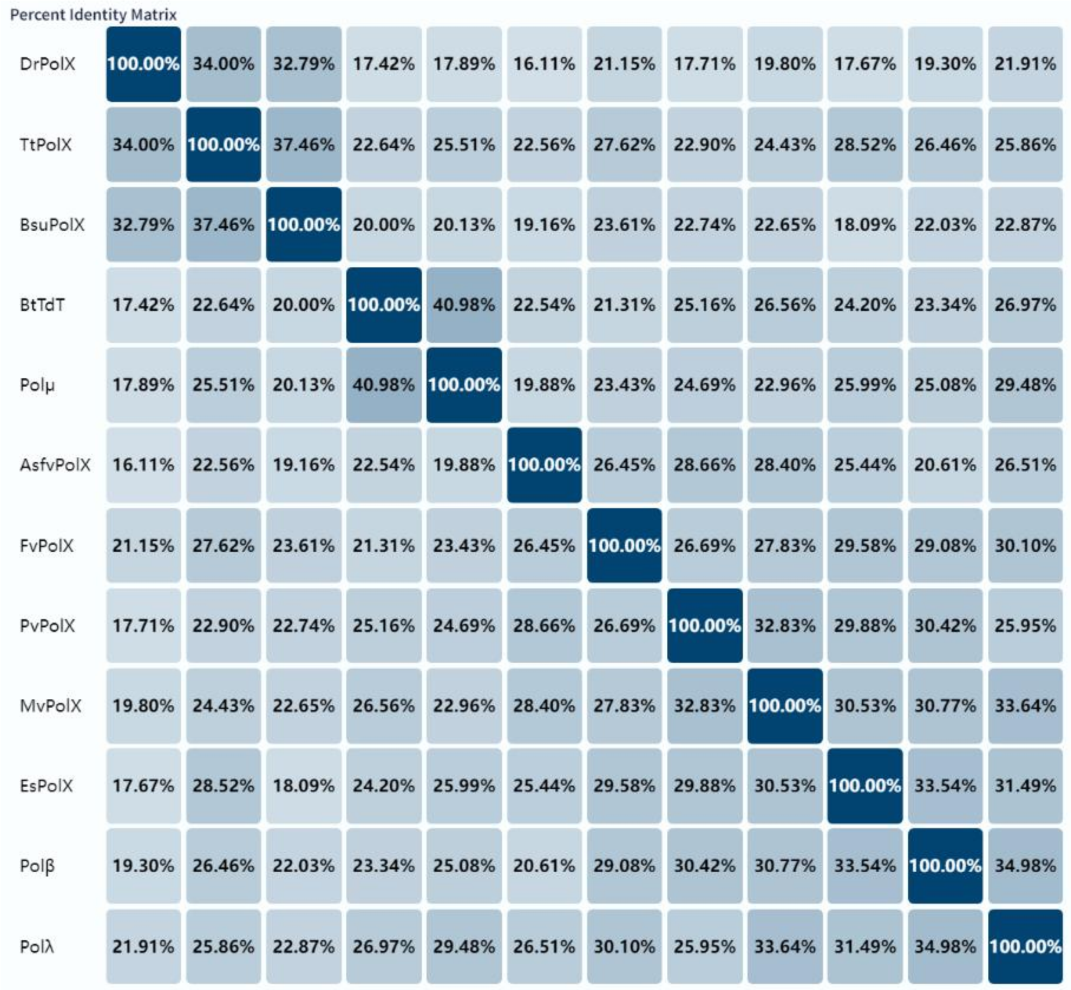

Percent Identity Matrix

DrPolX	100.00%	34.00%	32.79%	17.42%	17.89%	16.11%	21.15%	17.71%	19.80%	17.67%	19.30%	21.91%
TtPolX	34.00%	100.00%	37.46%	22.64%	25.51%	22.56%	27.62%	22.90%	24.43%	28.52%	26.46%	25.86%
BsuPolX	32.79%	37.46%	100.00%	20.00%	20.13%	19.16%	23.61%	22.74%	22.65%	18.09%	22.03%	22.87%
BtTdT	17.42%	22.64%	20.00%	100.00%	40.98%	22.54%	21.31%	25.16%	26.56%	24.20%	23.34%	26.97%
Polμ	17.89%	25.51%	20.13%	40.98%	100.00%	19.88%	23.43%	24.69%	22.96%	25.99%	25.08%	29.48%
AsfvPolX	16.11%	22.56%	19.16%	22.54%	19.88%	100.00%	26.45%	28.66%	28.40%	25.44%	20.61%	26.51%
FvPolX	21.15%	27.62%	23.61%	21.31%	23.43%	26.45%	100.00%	26.69%	27.83%	29.58%	29.08%	30.10%
PvPolX	17.71%	22.90%	22.74%	25.16%	24.69%	28.66%	26.69%	100.00%	32.83%	29.88%	30.42%	25.95%
MvPolX	19.80%	24.43%	22.65%	26.56%	22.96%	28.40%	27.83%	32.83%	100.00%	30.53%	30.77%	33.64%
EsPolX	17.67%	28.52%	18.09%	24.20%	25.99%	25.44%	29.58%	29.88%	30.53%	100.00%	33.54%	31.49%
Polβ	19.30%	26.46%	22.03%	23.34%	25.08%	20.61%	29.08%	30.42%	30.77%	33.54%	100.00%	34.98%
Polλ	21.91%	25.86%	22.87%	26.97%	29.48%	26.51%	30.10%	25.95%	33.64%	31.49%	34.98%	100.00%

Figure S1. Amino acid sequence identity analysis of PolX enzymes. Aligned using the UniProt website.

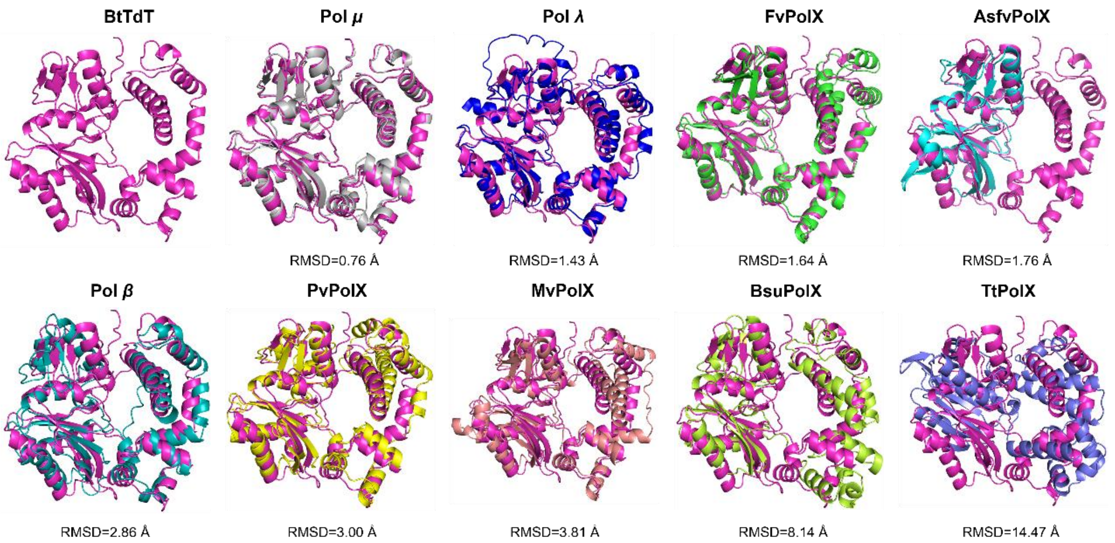

Figure S2. RMSD values of PolXc structures from different sources, aligned to the PolXc structure of the *Bos taurus* TdT (BtTdT). Structures were predicted by

AlphaFold3 (AF3) and aligned in PyMOL. Details of protein characteristics are provided in Supplementary Tables S1 and S2.

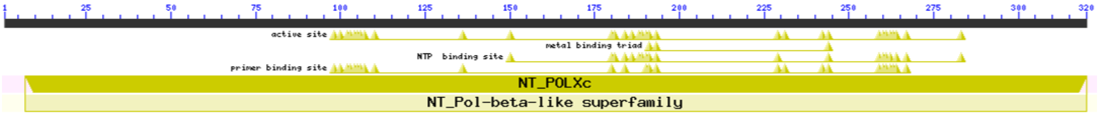

Figure S3. Conserved domains analysis of FvPolX in NCBI.

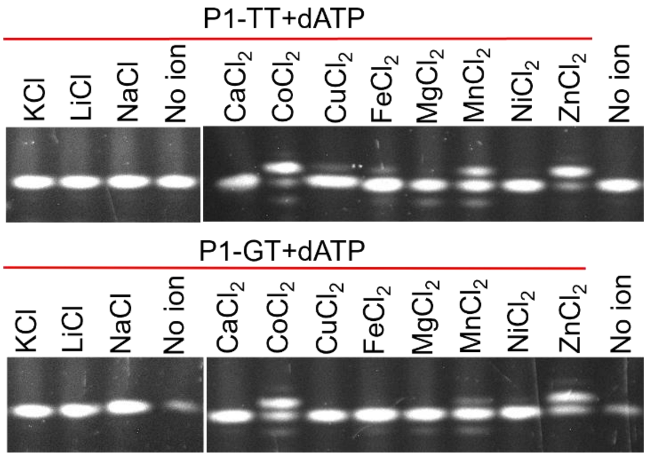

Figure S4. Denaturing ureaPAGE assays for FvPolX-catalyzed template-independent extension in presence of different metal ions. Reaction conditions: 0.5 mg/mL FvPolX, 1 μM iDNA, 30 °C for 10 min.

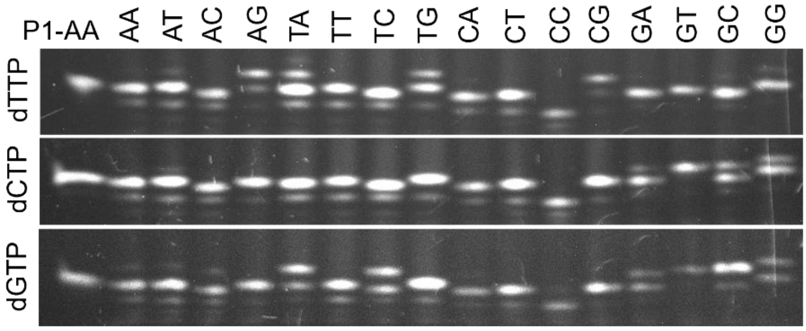

Figure S5. Activity analysis of FvPolX^{T186G} for dGTP, dCTP, or dTTP incorporation. Reaction conditions: 1 mg/mL FvPolX^{T186G}, 1 μM iDNA, 30 °C for 10 min with dGTP, dCTP, or dTTP.

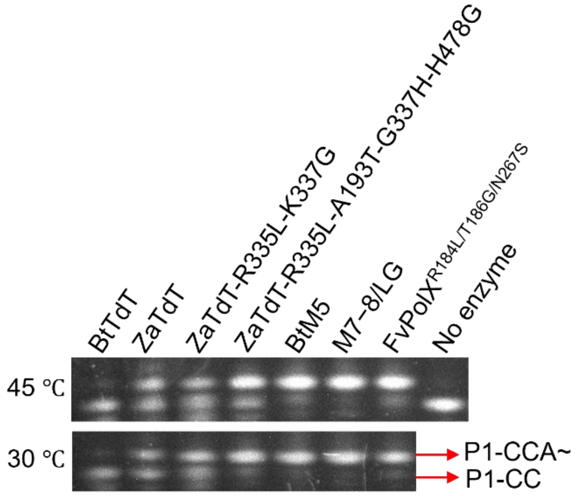

Figure S6. Activity comparison of FvPolX$^{R184L/T186G/N267S}$, wild-type TdTs and engineered TdT variants for adding 3′-ONH$_2$-dATP. Reaction conditions: 0.5 mg/mL enzyme, 1 μM iDNA P1-CC, 30 °C or 45 °C for 10 min with 3′-ONH$_2$-dATP. "A~" stands for 3′-ONH$_2$-dATP.

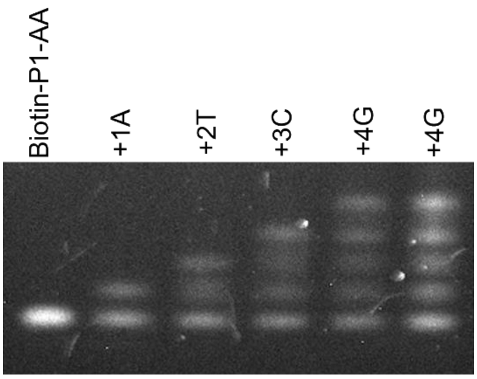

Figure S7. PAGE analysis of 4-nt oligonucleotide (ATCG) synthesis by FvPolX$^{R184L/T186G/N267S}$. Elongation reaction conditions: 1 mg/mL FvPolX$^{R184L/T186G/N267S}$, 1 μM preimmobilized iDNA biotin-P1-AA, 30 °C for 10 min with 3′-ONH$_2$-dATP, 3′-ONH$_2$-dTTP, 3′-ONH$_2$-dCTP, and 3′-ONH$_2$-dGTP, respectively at each synthesis step. Deblock reaction conditions: the reactions were treated with 0.7 M sodium nitrite buffer (pH 5) for 1 min at room temperature.

Table S1. PolX enzymes used in this study.

Protein	Species name	Class name	UniProt or NCBI ID[a]
FvPolX	*Faustovirus*	Viruse	A0A6M6DHZ3
AsfvPolX	*African swine fever virus*	Viruse	P42494
BtTdT	*Bos taurus*	Animalia	P06526
TtPolX	*Thermus thermophilus*	Bacteria	Q5SJ64
BsuPolX	*Bacillus subtilis*	Bacteria	P94544
DrPolX	*Deinococcus radiodurans*	Bacteria	Q9RX48
PvPolX	*Pithovirus*	Viruse	A0A481Z4U2
MvPolX	*Acanthamoeba polyphaga mimivirus*	Viruse	A0A0G2Y8D8
Pol β	*Homo sapiens*	Animalia	P06746
Pol λ	*Homo sapiens*	Animalia	Q9UGP5
Pol μ	*Homo sapiens*	Animalia	Q9NP87
EsPolX	*Eutrema salsugineum*	Plantae	XP_024008683.1

a: The sequences of the corresponding proteins are given in supplementary Table S2.

Table S2. Amino acid sequences of PolXs used in this study.

Enzyme	Amino acid sequence
FvPolX	MNNSPNREILAMLLEFAKIYDVLGDVFKSKAYTNAASTI<br>GKLSYALTRDNVEQFIASHPKGIGKGIASRIREFVESRRV<br>ADLDELKRSREYIAYTEFTKIIGVGPATARKWLEMGIDN<br>LLKLRQAVASGRVDLSHAQKLGLIYYADLNQRIPRAEVA<br>TIGAKVKSYINQLARDNKFEIVGSYRRGTQTSGDVDCVF<br>TTQKFDPKIIARLVEILETDTNFIDVLAQGEQKVTFLYRG<br>KATIRQVDVFYAAYPEYIPHILYATGSAEHNEYVRGEAK<br>RQGYRLNQIGLFKIGRNGAMKKITLHTERELYDILKIPYV<br>EPKDR
AsfvPolX	MLTLIQGKKIVNHLRSRLAFEYNGQLIKILSKNIVAVGSL<br>RREEKMLNDVDLLIIVPEKKLLKHVLPNIRIKGLSFSVKV<br>CGERKCVLFIEWEKKTYQLDLFTALAEEKPYAIFHFTGP<br>VSYLIRIRAALKKKNYKLNQYGLFKNQTLVPLKITTEKE<br>LIKELGFTYRIPKKRL
BtTdT	MDPLCTASSGPRKKRPRQVGASMASPPHDIKFQNLVLFI<br>LEKKMGTTRRNFLMELARRKGFRVENELSDSVTHIVAE<br>NNSGSEVLEWLQVQNIRASSQLELLDVSWLIESMGAGK<br>PVEITGKHQLVVRTDYSATPNPGFQKTPPLAVKKISQYAC<br>QRKTTLNNYNHIFTDAFEILAENSEFKENEVSYVTFMRA<br>ASVLKSLPFTIISMKDTEGIPCLGDKVKCIIEEIIEDGESSE<br>VKAVLNDERYQSFKLFTSVFGVGLKTSEKWFRMGFRSL<br>SKIMSDKTLKFTKMQKAGFLYYEDLVSCVTRAEAEAVG<br>VLVKEAVWAFLPDAFVTMTGGFRRGKKIGHDVDFLITSP<br>GSAEDEEQLLPKVINLWEKKGLLLYYDLVESTFEKFKLP<br>SRQVDTLDHFQKCFLILKLHHQRVDSSKSNQQEGKTWK<br>AIRVDLVMCPYENRAFALLGWTGSRQFERDIRRYATHER<br>KMMLDNHALYDKTKRVFLKAESEEEIFAHLGLDYIEPW<br>ERNA
TtPolX	MRNQELARIFEEIGLMSEFLGDNPFRVRAYHQAARTLYD<br>LDTPIEEIAEKGKEALMELPGVGPDLAEKILEFLRTGKVR<br>KHEELSRKVPRGVLEVMEVPGVGPKTARLLYEGLGIDSL<br>EKLKAALDRGDLTRLKGFGPKRAERIREGLALAQAAGK<br>RRPLGAVLSLARSLLEAIRALPGVERAELCGSARRYKDT<br>VGDLDFLVASREGERAVEGFVRLPQVKEVYAKGKERAT<br>VFLKNGLQVDLRVVPPESYGAGLQYLTGSKAHSIRLRAL<br>AQEKGLKLSEYGVFRGEKRIAGETEEEVYAALGLPWIPP<br>PLREDQGEVEAALEGRLPKLLELPQVKGDLQVHSTYSD<br>GQNTLEELWEAAKTMGYRYLAVTDHSPAVRVAGGPSPE<br>EALKRVGEIRRFNETHGPPYLLAGAEVDIHPDGTLDYPD<br>WVLRELDLVLVSVHSRFNLPKADQTKRLLKALENPFVH<br>VLAHPTARLLGRRAPIEADWEAVFQKAKEKGVAVEIDG<br>YYDRMDLPDDLARMAYGMGLWISLSTDAHQTDHLRFM

	ELAVGTAQRAWIGPERVLNTLDYEDLLSWLKARRGV
BsuPolX	MHKKDIIRLLETIAVYMELKGDNPFKVSAFRKAAAALE<br>QDDRSLSEMDDMMSLSGIGKGTYSVIKEYIDEGKSSTLE<br>SLQKEVPEGLVPLLKLPGLGGKKIAKLYKELGVHDAESL<br>KEACEQQKVQGLAGFGKKSEEKILQALGEAGKQPERFPI<br>GYALRIAREIEEHLSQFTHIIKFSRAGSLRRARETVKDLD<br>YIIATDHPAEVREQLLELPNIKSVIASGDTKVSVILSFEYE<br>TSVDFRLVTEEQFPTTLHHFTGSKDHNIKMRQIAKERGE<br>RISEYGVETVETGEIKTFPSEREFYAHFGLPLIPPEIRESGQ<br>EVETYSDSIELIELGQIKGDLHMHSTWSDGAFSIREMAE<br>ACIKKGYQYMAITDHSQYLKVANGLTAERLKQQAKEID<br>ALNAEFENFRILKGVEMDILPDGTLDYDDDVLAEMDIVI<br>ASIHSSFNQPEHVIMKRLETALTNKHVDIIAHPTGRLIGR<br>RAGYEIDIDQLIELARKTNTALELNANPARLDLRTEHLM<br>KANEQGVTLVINTDAHNIEMLDDMKTGVTAARKGWTE<br>TKNVLNARSLKDVEAFLKRND
DrPolX	MTLPPDAPSRHRLVHALERTADLLDILGGEDFKSRAYRS<br>AARSLEELNEETPELLAREFTGIPKVGKGIAAELSDFARS<br>GTFAPLEAAAGQLPPGLLDLLGVRGLGPKKIRSLWLAGI<br>DSLERLREAAESGELAGLKGFGAKSAATILENVVFLFEA<br>RQRQSLRAGLAVAEELAGALTDLSPAPAGDVRRGLETVR<br>AAELTVTGTPDDVLARLPELTVQGDGVLSGDYEGVPVEI<br>ACAPAEARGALDLLRSGEHFAGQVQAAAQARGFTLTAG<br>GLSRGDEVLPTPTEAVVFHALDLPFRPAEYREPEHDDLW<br>QTLPDPAELVTVGDLRGMIHTHSTWSDGGASIREMAEA<br>TLTLGHEFLGTADHSRAAYYANGLTIERLREQLKEIRELQ<br>RAGLPIVAGSEVDILDDGSLDFPDDVLGELDYVVVSVHS<br>NFTLDAARQTERLIRAVSHPLVTVLGHATGRLLLRRPGY<br>ALDLDAVLGACEANGTVVEINANAARLDLDWREALRW<br>RERLKFAINTDAHVPGGLRDARYGVMQARKAGLTPAH<br>VVNSLGRAEFLDFVARQRAARGAPGPADRA
PvPolX	MDIRKCIKSSSQGGLSRKEINEYAEKIGINFNNFRKKDDL<br>CDEIIRIEKIKELEVIKEIKNNELIIKTLNKLSSFYKKSGDK<br>FRSIAFSKAENLISKLNFNIEFIEQIKNIPNIGEGILKRIDEIL<br>KTGKLKELEQFGEDFDFTTILGFGPTHQRNLIDIGVKNIE<br>DLKDAISKKKFIPTILQNAGIKYLEDFKIKIPRSEIFDIGGL<br>IIKEAKKINPFIIIEIVGSYRRKKENSRDIDILISHKENVNIL<br>EELINNLMNIELIKEKLSLGKIKFMGIYFSNYMNKDLKPI<br>ARKIDIRFIPYESYHSALLHSTGPVDFNIKIRNIAITKNLSL<br>SEFGLLDKKSNIKYFIESEKNIFDILELTYLSPKDRENVIIL<br>K
MvPolX	MNSKIIEQFNLLEKQVDAEYLNSKVENDLKEETMNRFR<br>LKSIKKALSILKNLDFEITDANDVKGIPGIGAGTINRIKEI<br>LETGKLHDLKDKFSPEKQKQIEGIQELENVIGIGSSTAKK

	LVSQYGIRSVDDLKKAIETGKVKVSTSIMLGLKYYGIVQ<br>RDIPRKEITAIEKLLSKEAHKIDPDLEIIICGSYRRGKKTSG<br>DIDVLMYHPKMKTSKEMLHPEKFDLEPYFNLYIDQLTE<br>KGFLIDDITFNPNKKYMGFCKYKLNPVRRIDIRFIPYNSL<br>APAMLYFTGPMELNTKMRSAAKKRKMILNEYGLFKTD<br>KNGAQIPLDTKSEADIFHALGMDYLTPQQRELYSSGKIH
Pol β	MSKRKAPQETLNGGITDMLTELANFEKNVSQAIHKYNA<br>YRKAASVIAKYPHKIKSGAEAKKLPGVGTKIAEKIDEFL<br>ATGKLRKLEKIRQDDTSSSINFLTRVSGIGPSAARKFVDE<br>GIKTLEDLRKNEDKLNHHQRIGLKYFGDFEKRIPREEML<br>QMQDIVLNEVKKVDSEYIATVCGSFRRGAESSGDMDVL<br>LTHPSFTSESTKQPKLLHQVVEQLQKVHFITDTLSKGET<br>KFMGVCQLPSKNDEKEYPHRRIDIRLIPKDQYYCGVLYF<br>TGSDIFNKNMRAHALEKGFTINEYTIRPLGVTGVAGEPL<br>PVDSEKDIFDYIQWKYREPKDRSE
Pol λ	MDPRGILKAFPKRQKIHADASSKVLAKIPRREEGEEAEE<br>WLSSLRAHVVRTGIGRARAELFEKQIVQHGGQLCPAQG<br>PGVTHIVVDEGMDYERALRLLRLPQLPPGAQLVKSAWL<br>SLCLQERRLVDVAGFSIFIPSRYLDHPQPSKAEQDASIPPG<br>THEALLQTALSPPPPPTRPVSPPQKAKEAPNTQAQPISDD<br>EASDGEETQVSAADLEALISGHYPTSLEGDCEPSPAPAVL<br>DKWVCAQPSSQKATNHNLHITEKLEVLAKAYSVQGDK<br>WRALGYAKAINALKSFHKPVTSYQEACSIPGIGKRMAE<br>KIIEILESGHLRKLDHISESVPVLELFSNIWGAGTKTAQM<br>WYQQGFRSLEDIRSQASLTTQQAIGLKHYSDFLERMPRE<br>EATEIEQTVQKAAQAFNSGLLCVACGSYRRGKATCGDV<br>DVLITHPDGRSHRGIFSRLLDSLRQEGFLTDDLVSQEENG<br>QQQKYLGVCRLPGPGRRHRRLDIIVVPYSEFACALLYFT<br>GSAHFNRSMRALAKTKGMSLSEHALSTAVVRNTHGCK<br>VGPGRVLPTPTEKDVFRLLGLPYREPAERDW
Pol μ	MLPKRRRARVGSPSGDAASSTPPSTRFPGVAIYLVEPRM<br>GRSRRAFLTGLARSKGFRVLDACSSEATHVVMEETSAEE<br>AVSWQERRMAAAPPGCTPPALLDISWLTESLGAGQPVP<br>VECRHRLEVAGPRKGPLSPAWMPAYACQRPTPLTHHNT<br>GLSEALEILAEAAGFEGSEGRLLTFCRAASVLKALPSPVT<br>TLSQLQGLPHFGEHSSRVVQELLEHGVCEEVERVRRSER<br>YQTMKLFTQIFGVGVKTADRWYREGLRTLDDLREQPQK<br>LTQQQKAGLQHHQDLSTPVLRSDVDALQQVVEEAVGQ<br>ALPGATVTLTGGFRRGKLQGHDVDFLITHPKEGQEAGL<br>LPRVMCRLQDQGLILYHQHQHSCCESPTRLAQQSHMDA<br>FERSFCIFRLPQPPGAAVGGSTRPCPSWKAVRVDLVVAPV<br>SQFPFALLGWTGSKLFQRELRRFSRKEKGLWLNSHGLF<br>DPEQKTFFQAASEEDIFRHLGLEYLPPEQRNA
EsPolX	MAPKRGRNRSPSPDPAGMFAGMIWKQKLVQMGAVIEE

	NRLTKKVTHVLALNLEALLEKFGKESLSEFRGHFLLYQ WLEDSLTAGEKANEDLYVLKIDSEETEEPNKSLPEDSVS EDQPSTQKRTRYSPDAGDIVGVENQTNTQGSPNSPTSCS VPSTSANPGEGTAETPTSPQSESTSVYKPPDLNRNITEIFG KLINIYRALGDDRRSFSYYKAIPVIEKLPTKIESVDQLQH LPGIGKAMKDHIQEIVTTGKLSKLEHFETDEKVRTISLFG EVWGIGPATALKLYDKGHRTLEDLKNEDSLTHAQRLGL KYFDDIRTRIPRQEVQEMEQLLQRVGEEILPGVNIVCGGS YRRGKATCGDLDIVVTHPDGQSHKGFLTKFVKRLKDIN FLREDLIFSTHSEEGTDAGVDTYFGLCTYPGQELRRRIDF KVYPRDIYAFGLIAWTGNDVLNRRLRLLAESKGYRLDD TGLFPATHSSSGNRGARASASLKLSTEKQVFDFLGFPWL EPHERNL
FvPolX$^{R184L/T186G/N267S}$	MNNSPNREILAMLLEFAKIYDVLGDVFKSKAYTNAASTI GKLSYALTRDNVEQFIASHPKGIGKGIASRIREFVESRRV ADLDELKRSREYIAYTEFTKIIGVGPATARKWLEMGIDN LLKLRQAVASGRVDLSHAQKLGLIYYADLNQRIPRAEVA TIGAKVKSYINQLARDNKFEIVGSYRLGGQTSGDVDCVF TTQKFDPKIIARLVEILETDTNFIDVLAQGEQKVTFLYRG KATIRQVDVFYAAYPEYIPHILYATGSAEHSEYVRGEAKR QGYRLNQIGLFKIGRNGAMKKITLHTERELYDILKIPYVE PKDR
s149FvPolX$^{R184L/N267S}$	QRIPRAEVATIGAKVKSYINQLARDNKFEIVGSYRRGTQ TSGDVDCVFTTQKFDPKIIARLVEILETDTNFIDVLAQGE QKVTFLYRGKATIRQVDVFYAAYPEYIPHILYATGSAEHN EYVRGEAKRQGYRLNQIGLFKIGRNGAMKKITLHTERE LYDILKIPYVEPKDR
BtM5	KKISQYACQRKTTLNNYNHIFTDAFEILAENSEFKENEVS YVTFMRAASVLKSLPFTIISMKDTEGIPCLGDKVKCIIEEI IEDGESSEVKAVLNDERYQSFKLFTSVFGVGLKTSEKWF RMGFRSLSKIMSDKTLKFTKMQKAGFLYYEDLVSCVTR AEAEAVGVLVKEAVWAFLPDAFVTMTGGFRLGGKIGHD VDFLITSPGSAEDEEQLLPKVINLWEKKGLLLYYDLVEST FEKFKLPSRQVGTMDHFQKCFLILKLHHQRVDSSKSNQ QEGKTWKAIRVDLVMCPYENRAFALLGWTGSRQFSRDI RRYATHERKMMLDNHALYDKTKRVFLKAESEEEIFAHL GLDYIEPWERNA
s120FvPolX	LKLRQAVASGRVDLSHAQKLGLIYYADLNQRIPRAEVATI GAKVKSYINQLARDNKFEIVGSYRRGTQTSGDVDCVFT TQKFDPKIIARLVEILETDTNFIDVLAQGEQKVTFLYRGK ATIRQVDVFYAAYPEYIPHILYATGSAEHNEYVRGEAKR QGYRLNQIGLFKIGRNGAMKKITLHTERELYDILKIPYVE PKDR
M7-8/LG	MKVSQYACQRRTTLNNHNKRFTDAFEIMAEYYEFNENE

	GRCLAFRRAASVLKSLPFTVTRMKDIQGLPCFGDHVRRI<br>IQEILEHGESSEVERVLNDERYQAFKLFTSVFGVGVKTAE<br>KWYRMGLRTVEEVKADKTLKLTKMQKAGLQYYEDLV<br>SCVSKAEADAISQIVKETVWAFLPDALVTMTGGFRLGG<br>EIGHDVDFLITNPGPREDDELLHKVIDLWKKQGLLLYCD<br>IIESTFDKSKLPSRKVDAMDHFQKCFCILKLYQPRVDNST<br>YNTSKQLDMAEVKDWKAVRVDLVVTPYEQYAFALLGW<br>TGSKQFNRDLRRYARHERKMLLDNHGLYDRTQKIFLKA<br>TSEEEIFAHLGLEYIPPWERNA
ZaTdT-R335L-A193T-G337H-H478G	MDRFKAPAVISQRKRQKGLHSPKLSCSYEIKFSNFVIFIM<br>QRKMGLTRRMFLMELGRRKGFRVESELSDSVTHIVAEN<br>NSYLEVLDWLKGQAVGDSSRFELLDISWFTACMEAGRP<br>VDSEVKYRLMEQSQSLPLNMPALEMPAFIATKVSQYSC<br>QRKTTLNNYNKKFTDAFEVMAENYEFKENEIFCLEFLRT<br>ASLLKSLPFSVTRMKDIQGLPCVGDQVRDIIEEIIEEGESS<br>RVNEVLNDERYKAFKQFTSVFGVGVKTSEKWYRMGLR<br>TVEEVKADKTLKLSKMQKAGLLYYEDLVSCVSKAEAD<br>AVSLIVKNTVCTFLPDALVTITGGFRLGHNIGHDIDFLITN<br>PGPREDDELLHKVIDLWKKQGLLLYCDIIESTFVKEQLPS<br>RKVDAMDHFQKCFAILKLYQPRVDNSTCNTSEQLEMAE<br>VKDWKAIRVDLVITPFEQYPYALLGWTGSRQFGRDLRR<br>YAAHERKMILDNGGLYDRRKRIFLKAGSEEEIFAHLGLD<br>YVEPWERNA
ZaTdT-R335L-K337G	MDRFKAPAVISQRKRQKGLHSPKLSCSYEIKFSNFVIFIM<br>QRKMGLTRRMFLMELGRRKGFRVESELSDSVTHIVAEN<br>NSYLEVLDWLKGQAVGDSSRFELLDISWFTACMEAGRP<br>VDSEVKYRLMEQSQSLPLNMPALEMPAFIATKVSQYSC<br>QRKTTLNNYNKKFTDAFEVMAENYEFKENEIFCLEFLR<br>AASLLKSLPFSVTRMKDIQGLPCVGDQVRDIIEEIIEEGES<br>SRVNEVLNDERYKAFKQFTSVFGVGVKTSEKWYRMGL<br>RTVEEVKADKTLKLSKMQKAGLLYYEDLVSCVSKAEA<br>DAVSLIVKNTVCTFLPDALVTITGGFRLGGNIGHDIDFLIT<br>NPGPREDDELLHKVIDLWKKQGLLLYCDIIESTFVKEQL<br>PSRKVDAMDHFQKCFAILKLYQPRVDNSTCNTSEQLEM<br>AEVKDWKAIRVDLVITPFEQYPYALLGWTGSRQFGRDL<br>RRYAAHERKMILDNHGLYDRRKRIFLKAGSEEEIFAHLG<br>LDYVEPWERNA
ZaTdT	MDRFKAPAVISQRKRQKGLHSPKLSCSYEIKFSNFVIFIM<br>QRKMGLTRRMFLMELGRRKGFRVESELSDSVTHIVAEN<br>NSYLEVLDWLKGQAVGDSSRFELLDISWFTACMEAGRP<br>VDSEVKYRLMEQSQSLPLNMPALEMPAFIATKVSQYSC<br>QRKTTLNNYNKKFTDAFEVMAENYEFKENEIFCLEFLR<br>AASLLKSLPFSVTRMKDIQGLPCVGDQVRDIIEEIIEEGES<br>SRVNEVLNDERYKAFKQFTSVFGVGVKTSEKWYRMGL

	RTVEEVKADKTLKLSKMQKAGLLYYEDLVSCVSKAEA<br>DAVSLIVKNTVCTFLPDALVTITGGFRRGKNIGHDIDFLIT<br>NPGPREDDELLHKVIDLWKKQGLLLYCDIIESTFVKEQL<br>PSRKVDAMDHFQKCFAILKLYQPRVDNSTCNTSEQLEM<br>AEVKDWKAIRVDLVITPFEQYPYALLGWTGSRQFGRDL<br>RRYAAHERKMILDNHGLYDRRKRIFLKAGSEEEIFAHLG<br>LDYVEPWERNA

Table S3. Primers used in this study.

Primers	Sequence 5′ to 3′	Description
s149FvPolX-F	CAGCAGCCATCACCATCATCACCACC AGCGCATTCCGCGTGCGGAAGT	For cloning s149FvPolX
s149FvPolX-R	CTGTTCGACTTAAGCATTATGCTCAGC GATCTTTCGGCTCCACATA	For cloning s149FvPolX
s120FvPolX-F	CAGCAGCCATCACCATCATCACCACC TGAAACTGCGCCAGGCGGTGGC	For cloning s120FvPolX
s120FvPolX-R	CTGTTCGACTTAAGCATTATGCTCAGC GATCTTTCGGCTCCACATA	For cloning s120FvPolX
FvPolX-F	CAGCAGCCATCACCATCATCACCACAT GAACAACAGCCCGAACCGCG	For cloning FvPolX
FvPolX-R	CTGTTCGACTTAAGCATTATGCTCAGC GATCTTTCGGCTCCACATA	For cloning FvPolX
FvPolX^{T186G}-F	ATCGCCGCGGCGGCCAGACTAGCGGT GATGTTGATTG	For cloning FvPolX^{T186G}
FvPolX^{T186G}-R	CCGCTAGTCTGGCCGCCGCGGCGATA GCTGCCCACAA	For cloning FvPolX^{T186G}
FvPolX^{R184L}-F	GCAGCTATCGCCTGGGCACCCAGACT AGCGGTGATGT	For cloning FvPolX^{R184L}
FvPolX^{R184L}-R	GTCTGGGTGCCCAGGCGATAGCTGCC CACAATTTCAA	For cloning FvPolX^{R184L}
FvPolX^{N267S}-F	GCGCGGAACATAGCGAATATGTGCGC GGCGAAGCGAA	For cloning FvPolX^{N267S}
FvPolX^{N267S}-R	CGCACATATTCGCTATGTTCCGCGCTG CCGGTCGCAT	For cloning FvPolX^{N267S}
P1	TAATACGACTCACTA	Substrate iDNA
P1-AA	TAATACGACTCACTAAA	Substrate iDNA
P1-AT	TAATACGACTCACTAAT	Substrate iDNA
P1-AC	TAATACGACTCACTAAC	Substrate iDNA
P1-AG	TAATACGACTCACTAAG	Substrate iDNA
P1-TA	TAATACGACTCACTATA	Substrate iDNA
P1-TT	TAATACGACTCACTATT	Substrate iDNA
P1-TC	TAATACGACTCACTATC	Substrate iDNA
P1-TG	TAATACGACTCACTATG	Substrate iDNA
P1-CA	TAATACGACTCACTACA	Substrate iDNA
P1-CT	TAATACGACTCACTACT	Substrate iDNA
P1-CC	TAATACGACTCACTACC	Substrate iDNA
P1-CG	TAATACGACTCACTACG	Substrate iDNA
P1-GA	TAATACGACTCACTAGA	Substrate iDNA
P1-GT	TAATACGACTCACTAGT	Substrate iDNA
P1-GC	TAATACGACTCACTAGC	Substrate iDNA
P1-GG	TAATACGACTCACTAGG	Substrate iDNA
